# His-tag ELISA for the detection of humoral tumor-specific immunity

**DOI:** 10.1186/1471-2172-9-23

**Published:** 2008-05-29

**Authors:** Vivian Goodell, Douglas McNeel, Mary L Disis

**Affiliations:** 1 Center for Translational Medicine in Women's Health, University of Washington, Seattle, WA 98109-8050, USA; 2 School of Medicine and Public Health, University of Wisconsin, Madison, WI 53792, USA

## Abstract

**Background:**

The application of high throughput molecular techniques such as SEREX are resulting in the identification of a multitude of tumor associated antigens. As newly identified antigens are incorporated into a variety of clinical trials, standardization of immunologic monitoring methods becomes increasingly important. We questioned whether mammalian cell expression of a histadine-linked human protein could be used to produce antigen suitable for detecting tumor-specific humoral immunity and whether such an assay could be amenable to standardization for clinical use.

**Methods:**

We designed a his-tagged capture ELISA based on lysate from genetically engineered CHO cells for detection of antibodies to insulin-like growth factor binding protein 2, a novel tumor antigen. We performed technical and preliminary clinical validation studies, including comparison to a standard indirect ELISA based on commercially prepared recombinant antigen.

**Results:**

The his-tagged capture ELISA could be standardized. Precision experiments resulted in CVs < 15%. Linearity and calibration experiments demonstrated r^2 ^values of 0.99. In comparison to Western blot analysis, his-tag and indirect ELISA accurately identified 88% and 93% of samples, respectively. Sample concordance between capture and indirect assays was highly significant (p = 0.003). Furthermore, significantly greater levels of IGFBP-2 antibody immunity were found in cancer patients compared to normal controls (p = 0.008).

**Conclusion:**

A genetically engineered cell lysate based ELISA can be amenable to standardization and can detect increased levels of antibody immunity to tumor-associated antigen in cancer patients compared to non tumor-bearing healthy controls.

## Background

Applications of high-throughput molecular techniques are resulting in the identification of a multitude of tumor associated antigens. Genomic and proteomic technologies have allowed immunogenic proteins to be determined for a wide variety of cancers. Both approaches utilize humoral immune responses to screen for tumor-associated antigens. Serological analysis of recombinant cDNA expression libraries has led to the identification of hundreds of cancer-specific antigens using sera from cancer patients to probe proteins encoded by tumor cDNA libraries. Several of the proteins identified by this technique have proven specific enough, and immunogenic enough, to be incorporated into tumor vaccines currently in clinical trials [1,2].

Specificity and immunogenicity of the many candidate vaccine antigens identified must be comprehensively characterized through population-based and laboratory studies prior to initiation of clinical trials. The quality of assays used for this purpose must meet standards mandated by the Clinical Laboratory Improvement Act (CLIA) in order to make assessment of clinical trials meaningful and to allow for comparison between trials conducted by different groups. High-throughput, rapid screening assays generally rely on commercially prepared and quality-controlled proteins. However, many newly identified tumor-associated antigens are not commercially available, and due to expense, limited demand, and difficulties involved in mass production of purified product, may not be produced outside the research lab. Given the need for both high quality and flexibility in assay methods, we questioned whether his-tagged capture ELISA could be utilized for evaluating antibody immunity against novel antigens. If such an assay could be optimized to meet clinical laboratory standards, it could serve as a template method for use with potentially any cDNA of interest.

The hexahistadine tagging of recombinant protein expressed in a transfected cell line offers several important advantages to the development of antibody screening assays. The method is relatively inexpensive compared to preparation of recombinant protein. Moreover, post translational modification necessary for immune recognition is maintained by choosing a eukaryotic cell line for protein expression.

We have recently established insulin-like growth factor binding protein (IGFBP-2) as a human tumor antigen found at elevated levels in colon and breast cancer patients [3], and in colon cancer patients IGFBP-2 overexpression is highly correlated with metastases and recurrence [4]. Furthermore, overexpression of the IGFBP-2 gene is associated with multi-drug resistance in human colon carcinoma cell lines [5]. As recombinant IGFBP-2 protein is commercially available, the antigen serves as a unique model in which to determine the clinical utility of his-tagged ELISA.

## Methods

### Subjects

Serum from 80 breast cancer patients and 80 colorectal cancer patients of any stage of disease and either sex was obtained after IRB approval and informed consent for the analysis of immunity against cancer. Patients had to be greater than 30 days from the last cytoreductive chemotherapy. Age range of the patient group was 36–91 years. The reference population sera was derived from non-cancer bearing volunteers, n = 200, contributing blood products at the Puget Sound Blood Center, Seattle, WA, aliquoted into 200 ul fractions, and stored at -70°C. Individuals contributing the sera samples met all the health requirements associated with blood donation. Age range of the serologic control group was 18–72 years, 106 samples were from men and 94 samples were from women.

### Vector construction and CHO cell transfection

The vector containing the cDNA for IGFBP-2 (a gift from Dr. S. Plymate, University of Washington) and the pcDNA4/HisMax B (Invitrogen, Carlsbad, CA) vector containing the histadine tag and Zeocin resistance gene were cut by digest with EcoRI and NotI. The full-length coding sequence was ligated in frame to the HisMax B vector according to commercial protocol (Invitrogen). Briefly, 0.5 ul HisMax B vector, 26 ul IGFBP-2 insert, 0.5 ul 2× Ligation Master Mix (Qiagen, Valencia, CA), and 3 ul distilled H_2_O were mixed and incubated at 16°C for 2 hours. The ligation/reaction mixture was stored at -20°C. The coding region was confirmed by DNA sequencing.

The HisMax construct containing IGFBP-2 was then used to transfect CHO cells as described below. CHO cells were prepared for transfection by seeding 6-well plates with 0.5 × 10^6 ^CHO cells/well in RPMI with 10% FCS and incubated at 37°C in 5% CO_2 _overnight. Cells were then washed and 1.5 ml of media was added to each well.

Two ug of the HisMax B/IGFBP-2 vector were diluted with EC buffer (Qiagen) to a total volume of 100 ul. The diluted vector was then vortexed for 1 second and incubated at room temperature for 5 minutes after addition of 32 ul of Enhancer (Qiagen). Ten ul of Effectene Transfection Reagent (Qiagen) was added to the DNA mixture and mixed by vortexing, and incubated at room temperature for 10 minutes. After addition of 0.5 ml media to the DNA mixture, the DNA mixture was added to the plated cells by pipetting up and down. Plates were then incubated at 37°C in 5% CO_2 _for 2 days. Cells were transferred to flasks and kept in culture in RPMI 10% FCS to which 150 ug/ml Zeocin (InvivoGen, San Diego, CA) was added as selection agent. As a control, another plate of cells was identically transfected with the pcDNA4/HisMax C vector without the IGFBP-2 insert.

### Preparation of cell lysate as source of protein

Cells transfected with the his-tagged IGFBP-2 or the tagged mock-insert (His-B) were harvested at log-phase growth by scraping. Cells were centrifuged and kept at -70°C until sufficient cells had accumulated to prepare 50 ml of lysate. Cells were then thawed and incubated in lysate buffer consisting of Tris base, NaCl and Triton-X, with aprotinin (1 ug/ml), benzamidine (1 mmol/L) and phenylmethylsulfonyl fluoride (1 mmol/mL) added. Cells were added to lysis buffer at a 10^8 ^cells/ml buffer concentration, kept on ice and vortexed every 10 minutes for 1 hour. Cells were then centrifuged for 20 minutes at -20°C, supernatant was collected, aliquoted, and stored at -70°C. The protein content of each lot of supernatant was quantified (BioRad colorimetric Protein Assay kit #500-0116), and protein identity confirmed for each lot by Western blot analysis using a goat polyclonal anti-IGFBP-2 antibody (Santa Cruz Biotechnology, Santa Cruz, CA) as primary antibody and rabbit anti-goat-HRP conjugate (Zymed, S. San Francisco) as secondary antibody. We used a low molecular weight ladder (BioRad, Hercules, CA) and a sample of human recombinant IGFBP-2 (Research Diagnostics, Inc, Flanders, NJ) as positive controls and a sample of the mock-transfected CHO lysate as a negative control. Protein was immunoprecipitated by adding mouse monoclonal anti-IGFBP-2 antibody (Diagnostic Systems Laboratories, Webster, TX) to IGFBP-2/His-B cell lysate with Protein A+G and allowing incubation overnight at 4°C. The lysate was then vortexed and the pellet washed twice with NNET buffer and twice with NET buffer. SDS-PAGE running buffer was added after the last wash, and the pellet was stored at -4°C.

Another immunoprecipitation was prepared with patient serum by adding Protein G to IGFBP-2/His-B cell lysate and incubating on ice for 5 minutes, vortexing several times. The supernatant was collected and incubated overnight at 4°C after the addition of Protein A+G and patient serum positive by ELISA for IGFBP-2 antibodies. The pellet was prepared as described above.

### Development of ELISA with protein derived from engineered CHO lysate

Microtiter plates (Dynex Technologies, Inc., Chantilly, VA) were coated overnight with his-tag specific monoclonal antibody (Qiagen) diluted 1/1000 in carbonate buffer. Only Immulon 4 HBX plates from a single lot were used throughout the validation. The standard curve was prepared by adding purified human IgG titrated to range from 0.62 ug/ml to 0.005 ug/ml, 50 ul/well, to the last column. All IgG for the standard curves was aliquoted from the same vial to assure consistency between curves. Plates were blocked with 100 ul PBS/5% BSA for 8 hours at room temperature, washed 4 times with PBS/0.01% tween-20 and alternating columns coated with 50 ul IGFBP-2 lysate diluted to 80 ug/ml in PBS/BSA, the mock vector, or PBS/BSA buffer alone. Plates were then incubated overnight at 4°C. Following 4 washes with PBS/Tween, patient sera was added after dilution with 10% NGS/10% FCS/PBS/1% BSA/25 1:25 through 1:200 and incubated 2 hours at room temperature. Plates were washed and goat IgG-HRP conjugate (Zymed Laboratories, San Francisco, CA) diluted 1:50,000 in PBS/BSA buffer, 50 ul/well, was added and incubated for 45 minutes at room temperature. Plates were washed 4 times and developed with 75 ul TMB reagent (Kirkegaard and Perry Laboratories, Gaithersburg, MD) and read at 640 nm. Reaction was stopped with 75 ul 1 N HCL when the 0.16 μg/ml standard reached an OD of 0.3, and plates were read at 450 nm. The OD of each serum dilution was calculated as the OD of the lysate-coated wells minus the OD of the His B-coated wells.

Positive control serum was run on every plate, as was a negative control of PBS/BSA buffer treated as a serum sample. Results for the IGFBP-2 antibody capture ELISA assay were confirmed by Western blot analysis. Samples were designated positive by ELISA based on a non-parametric 95^th ^percentile cut-point obtained by running 200 normal control samples through the assay. Positive cut-point for the capture assay was 0.18 ug/ml (0.024+/-0.051).

### Development of ELISA with purified recombinant IGFBP-2

In order to address the issue of anti-histidine or anti-nickel interference in the capture assay, to and compare capture assay results to the gold standard ELISA, we developed an indirect ELISA for IGFBP-2 antibodies. Immulon 4HBX microtiter plates (Dynex), were coated overnight with 50 ul of highly purified, human recombinant IGFBP-2 protein (Sigma Chemical Co. St. Louis, MO) diluted 1/1000 with carbonate buffer to a concentration of 0.5 ug/ml, or carbonate buffer alone in alternating columns. The last column of wells was incubated with serially diluted, purified human IgG (Sigma) to provide a standard curve.

Plates were blocked with 100 ul 1% BSA/PBS at room temperature for 1 hour, washed 4 times with PBS/Tween-20 and coated with experimental serum titrated as above. Plates were incubated for 1 hour at room temperature, washed 4 times and incubated for 45 minutes at room temperature after addition of 50 ul IgG-HRP conjugate (Zymed). Plates were washed, TMB was added and plates were developed and read as above. The OD of each serum dilution was calculated as the OD of the protein-coated wells minus the OD of the buffer-coated wells. Values for ug/ml were calculated from the standard curve on each plate, and positive cut-point for the indirect ELISA was set at 0.5 ug/ml (0.026+/-0.148).

### Assay validation

Standardization and validation of the capture ELISA was conducted as previously reported [6]. Briefly, accuracy was determined by assay of purified human IgG, diluted to 8 aliquots ranging from 0.62 μg/ml to .00025 μg/ml. The mean and CV for each concentration was compared to the expected value using regression analysis. The resulting r^2 ^value was used to quantify accuracy. Precision was determined by evaluating positive control samples in sets of 20 replicates. The 20 replicates were assayed together during one run to obtain the CV value for quantifying intra-assay precision. The 20 replicates were aliquoted, kept frozen and assayed individually during 20 separate runs over 6 weeks to obtain the CV value for quantifying interassay precision. Linearity and range were established by repeated assay of serially diluted positive samples, and quantified by the r^2 ^from regression analysis of expected versus resulting value. Sensitivity and specificity were determined by Chi^2 ^comparison of ELISA-positive and negative samples to the same samples assessed for IGFBP-2 antibodies by Western blot. A population of 200 normal donors, 80 breast and 80 colorectal cancer patients was evaluated to determine the ability of the capture assay to discriminate between tumor bearing and non-tumor bearing individuals.

### Statistical Analysis

All data were analyzed using SPSS 13.0 for Windows (SPSS Inc, Chicago, IL). Validation standards in linearity, range and accuracy were assessed by linear regression. Validation standards in sensitivity and specificity were assessed by Chi^2 ^table of Western blot and dichotomized ELISA results. Differences in response to the mock vector were assessed by t-test. Presence of antibodies in patients and normal controls was analyzed by Chi^2 ^test, and magnitude of antibody responses was evaluated by Kruskal-Wallis and Mann-Whitney U test where appropriate. The relationship between sample results from the indirect assay and the capture assay was investigated by Pearson's product-moment correlation. Receiver operating characteristic (ROC) curves were constructed as a preliminary investigation of capture ELISA discriminatory capacity, and the area under the curve analyzed by Chi^2 ^test. All statistical tests were 2-tailed and corrected for multiple comparisons by Bonferroni adjustment where appropriate.

## Results

### Capture ELISA for measurement of antibody response to his-tagged tumor-associated antigen can be developed to meet clinical validation standards in linearity and precision

**Figure 1:**
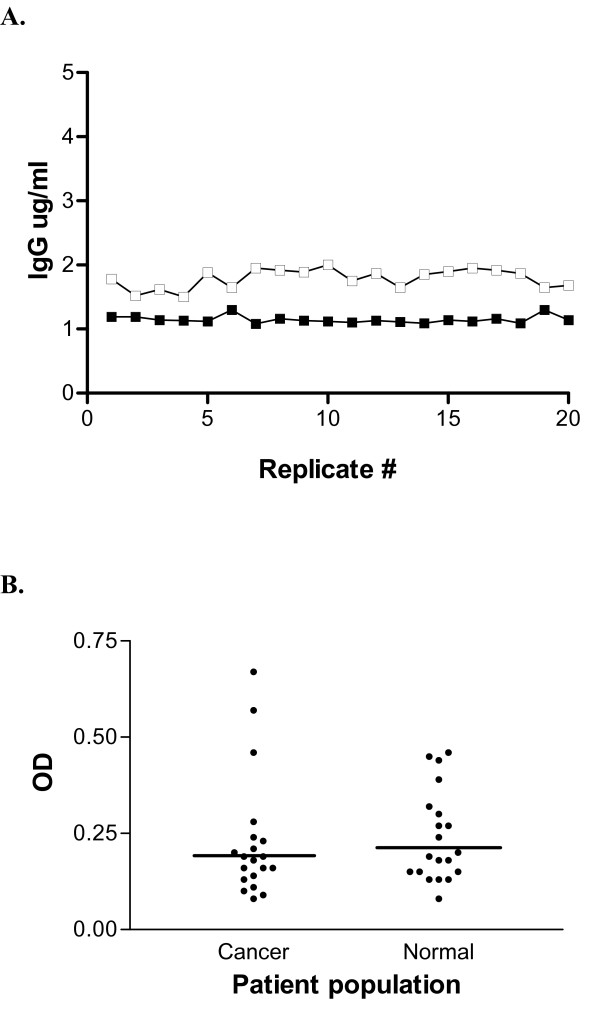
**Capture ELISA for measurement of antibody response to his-tagged tumor-associated antigen can be developed to meet standard requirements in precision, and there is no significant difference in antibody response to the his-tagged mock vector between normal donors or cancer patients**. (A) Twenty replicates of a sample positive for antibodies to IGFBP-2 were analyzed by capture ELISA to determine intra-assay precision (closed boxes) and interassay precision (open boxes). Data is expressed as IGFBP-2 IgG in ug/ml. (B) Response to the CHO cell lysate transfected with the his-tagged mock vector was assayed by capture ELISA for normal controls and cancer patients. Data is expressed as OD; bars represent mean OD for each group.

The standard required of a calibration curve for a clinical laboratory assay is an r^2 ^value of no less than 0.95, with a mean CV for replicate samples no greater than 15%. Validation of a lysate-based capture ELISA has been previously described in detail [6]. Similar calibration experiments showed that the mean CV for replicate samples (n= 20) measured by the IGFBP-2 capture ELISA was 8% and the r^2 ^for the assay was 0.99 (y = 0.001 + 0.815x, S_y/x _= 0.001). Sample linearity was also assessed with a resulting r^2 ^of 0.99 (y = 1.627 - 0.057x, S_y/x _= 0.025). Precision characteristics for the assay were established for single plate, single run (intra-assay) and between multiple runs (interassay). The capture assay of 20 positive replicates on a single plate yielded a mean of 1.16 ug/ml and CV of 13%. Multiple run precision was established over 20 consecutive experiments and returned a mean of 2.21 ug/ml and CV of 14%, below the standard maximum of 15% (Fig. 1A).

### There is no significant difference in antibody response to the his-tagged mock vector between controls and cancer patients

To address the possibility of differences in response to the empty vector between cancer patients and normal controls, we assayed 20 non-cancer bearing volunteers and 20 cancer patients by capture ELISA with the empty vector and compared the resulting mean optical densities. The Kolmogorov-Smirnov test for normality indicated approximately normal distribution, and the t-test found no significant difference (p = 0.770) between mean OD for the cancer group (OD = 0.24+/-0.118) and mean OD for the control group (OD = 0.24+/-0.159) (Fig. 1B).

### Capture ELISA results confirmed by Western blot analysis

**Figure 2:**
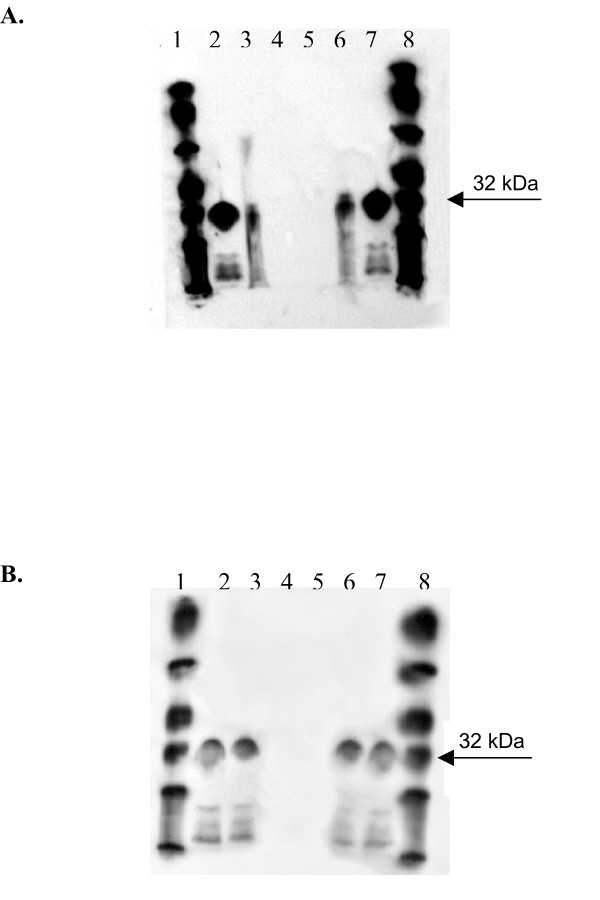
**Results from capture ELISA are confirmed by Western blot analysis**. (A) Representative example of a sample positive by capture ELISA analyzed by Western blot using IGFBP-2 lysate. Molecular weight (lanes 1&8), Western blot control (lanes 2&7), Immunoprecipitated IGFBP-2 lysate (lanes 3&6), and immunoprecipitated His-B lysate (lanes 4&5). (B) The same sample is also positive by Western blot using recombinant IGFBP-2 protein instead of hist-tagged protein. Molecular weight (lanes 1&8), Western blot control (lanes 2&7), recombinant human IGFBP-2 (lanes 3&6), and immunoprecipitated His-B lysate (lanes 4&5).

The cell lysate used as the capture ELISA antigen was immunoprecipitated with a histadine specific antibody, and the resulting immunoprecipitate run in Western blot analysis against 6 patient samples which assayed positive for IGFBP-2 antibodies by ELISA and 10 samples which assayed negative for IGFBP-2 antibodies by ELISA (Fig 2A). Identical blots were performed using commercially prepared recombinant human IGFBP-2 instead of the precipitated his tagged IGFBP-2 (Fig. 2B) in order to confirm the identity of the antigen recognized by antibodies in patient samples. A molecular weight ladder was run with each blot, as was a commercial IGFBP-2 Western blot positive control. The His-B lysate was immunoprecipitated with the histidine antibody and run as a negative control. Comparison of capture ELISA results to Western blot results for the same samples indicated that 88% of samples were correctly identified by ELISA. Thus, technical specificity was 83% and technical sensitivity was 100%.

### IGFBP-2 capture ELISA can discriminate between cancer patients and controls

**Figure 3:**
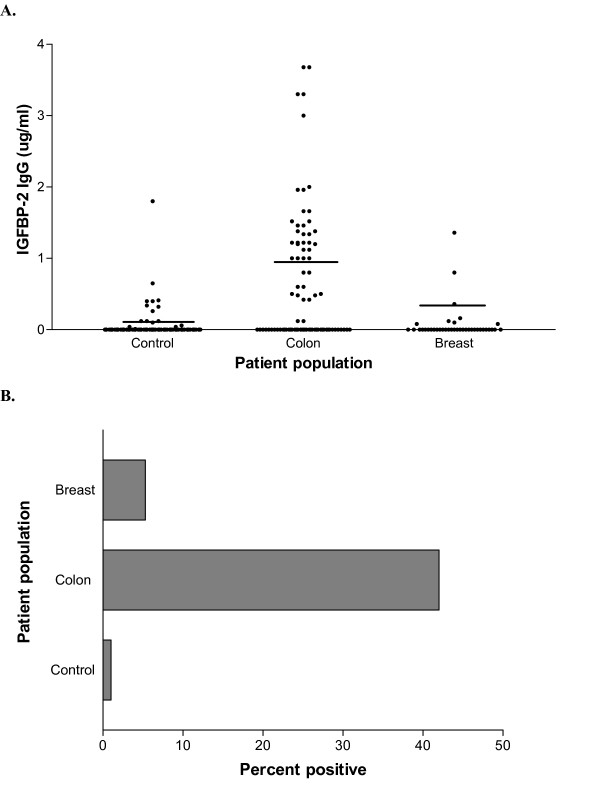
**The IGFBP-2 capture ELISA can discriminate between cancer patients and controls**. (A) Data is expressed as IGFBP-2 IgG in ug/ml. Lines represent mean of antibody responses to IGFBP-2 by capture ELISA. (B) Bars represent percentage of samples positive for antibodies to IGFBP-2 by capture ELISA.

Two hundred samples from healthy volunteers, 80 samples from breast cancer patients, and 80 samples from colon cancer patients were assayed for antibodies to IGFBP-2 using the capture ELISA. Magnitude of antibody response was significantly increased in breast and colon cancer patients compared to control samples (Fig. 3A) (p < 0.001) when 3-way analysis of continuous data was performed by Kruskal-Wallis test. Levels of IGFBP-2 antibodies were increased in breast cancer patients compared to normal volunteers (p = 0.013), and in colon cancer patients compared to normal volunteers (p < 0.001) when analyzed by Mann-Whitney U test. Continuous results from all samples were dichotomized using the 0.18 ug/ml cut-point derived from the reference range and subjected to Chi^2 ^test. The capture assay found that the presence of antibodies to IGFBP-2 was significantly increased in the population of cancer patient samples, where 23% of patients were positive, compared to control samples, with a positive rate of only 1% (p = 0.008). Furthermore, in comparisons between controls and each type of cancer, 5% of breast cancer patients (p = 0.032) and 40% of colorectal cancer patients (p < 0.001) had IGFBP-2 specific antibodies, compared to controls (Fig. 3B).

### There is significant concordance between patient sample results determined by his-tag capture ELISA and standard indirect ELISA

**Figure 4:**
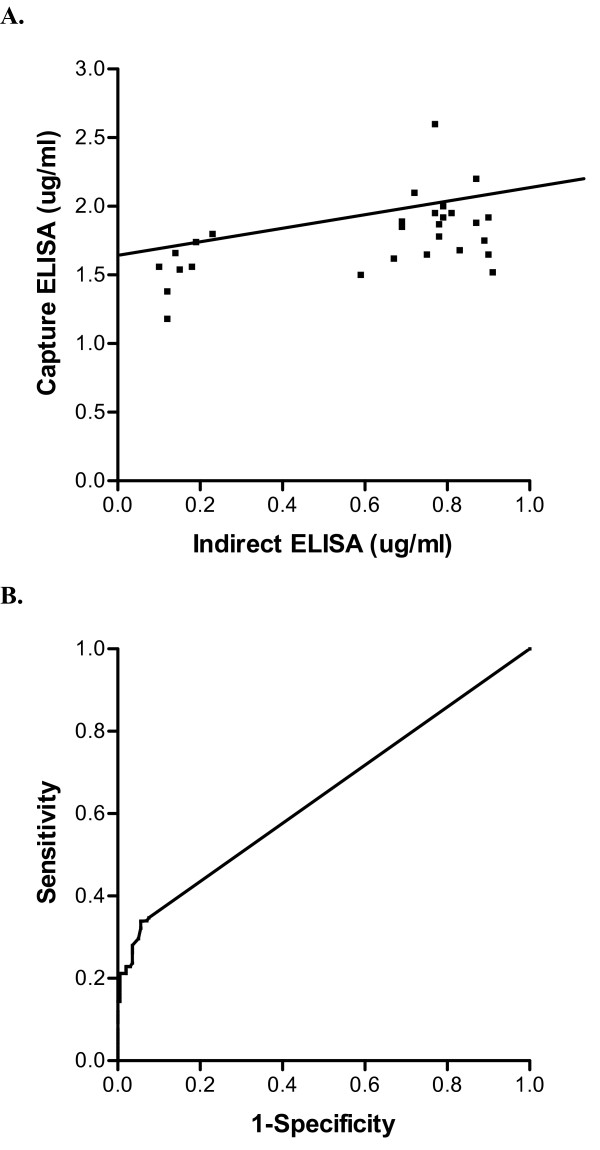
**Correlation of patient sample results by capture ELISA and indirect ELISA is significant**. (A) Results from samples positive by indirect ELISA (horizontal axis) and capture ELISA (vertical axis) were plotted and the strength of the association between results using both methods was assessed by Pearson's product-moment correlation. Results are in ug/ml, and the best-fit line is shown for reference. (B) The ROC curve was constructed using results from normal donors and patients with breast or colon cancer and plotted as diagnostic sensitivity vs. 1- diagnostic specificity.

We developed an indirect ELISA using commercially available recombinant human IGFBP-2. This assay meets all clinical laboratory requirements and allows comparison of a standard indirect ELISA with the his-tag-based capture ELISA described here (Table 1). The relationship between level of antibody response to the his-tagged IGBFP-2 protein by capture ELISA and level of antibody response by indirect ELISA was measured using Pearson's product-moment correlation (Fig. 4A). Matched results for all patient samples measured by both assay methods (n = 80) revealed a strong (r = 0.537), significant (p = 0.003) positive correlation. Preliminary assessment of the discriminatory capability of the assay was represented by ROC curves, and indicated that the capture ELISA is a weak predictor of presence of cancer with an area under the curve of 0.643 +/- 0.034, significantly different from the area expected by chance (p < 0.001) (Fig. 4B).

**Table 1:** Direct and his-tag ELISAs for IGFBP-2 antibodies meet validation standards.

**Parameter evaluated**	**Assay for antibodies to IGFBP2**		**Standard**
		
	**Indirect**	**His-tag**	
Limit of detection, ug/ml	0.0005	0.0005	N/A
Intra-assay cv	12%	13%	<15%
Inter-assay cv	11%	14%	<15%
Accuracy	93%	88%	
(technical specificity)	(91%)	(83%)	>90%
(technical sensitivity)	(100%)	(100%)	
Calibration curve	r^2 ^= 0.99	r^2 ^= 0.99	r^2 ^= 0.95
	cv = 7%	cv = 8%	cv <15%
Reference range, ug/ml	0.025+/-0.148	0.024+/-0.051	N/A

## Discussion

Many new tumor antigens are being identified. There is a need to develop rapid, accurate methods to assess immunity to novel antigens in large numbers of samples. We questioned whether a capture ELISA using engineered protein could be constructed to be reproducible to clinical standards. The use of tumor cell lysates as a source for antigen avoids many of the issues associated with developing high throughput assays for humoral immune monitoring such as lack of commercially prepared proteins and background responses to *E. coli *used in producing recombinant proteins [7,8]. We have previously shown that tumor cell lysate-based assays can be made CLIA-ready and suitable for high-throughput use in the clinical lab [6]. Data presented here demonstrates eukaryotic cell lines engineered to express tumor antigens can be developed to meet clinical laboratory specificities. Clinical grade his-tagged ELISA will greatly facilitate analysis of cancer specific humoral immunity.

The use of human tumor cell lines as a source of protein presents unique challenges. Mammalian cells, such as human tumor cells, have complex nutritional and environmental requirements compared to prokaryotic cells, making the culture of these cells complex, costly and prone to contamination. Lack of a cell wall, such as that found in bacteria, makes human cell lines more vulnerable to mechanical damage and may impact integrity of the protein of interest. Furthermore, proteins may naturally be expressed at only low levels in the cell. This may be rectified by induction of increased expression via reagents or equipment such as bioreactors or microcarriers [8], adding to the complexity and expense of high-yield protein production. In addition, high background responses associated with human cells may prevent detection of marginal immune responses to tumor antigens. Unlike responses to infectious disease antigens, antibodies to tumor-associated proteins are likely to be modest in magnitude and of lower affinity [9]. Most importantly, proteins derived from tumor cells are necessarily limited to those proteins found naturally in the cell line [10,11]. The identification of a cell line which overexpresses a particular protein can be a laborious, time-consuming and potentially an unsuccessful process.

With the widening availability of cDNA libraries and current ease of cloning techniques, genes encoding proteins of interest to tumor antigen discovery efforts are accessible and easy to handle. The use of CHO cells and the hexahistadine tag offers key advantages to the development of tumor-associated antigen assays. Mammalian cell processing preserves post-translational modification which has been shown to be important to immune recognition [8,10], and CHO cells, cultured in laboratories since the early 1960s, have demonstrated the relatively rapid, robust growth and relatively high protein expression required. CHO cells have proven capable of producing naturally-occurring proteins, transfected animal proteins and human recombinant proteins [12,13].

## Conclusion

Our experience with the IGFBP-2 transfected CHO line demonstrates that the cells can be kept in culture for up to 3 years without loss of protein expression, and that quantity and quality of the his-tagged protein remains consistent over that time. The his-tagged antigen also provides a means of purifying the protein during assay, eliminating the need for extra purification steps with associated cost and time. By coating ELISA plates with an anti-histadine monoclonal antibody prior to application of protein, the antigen presented to the experimental sample is limited to the tagged protein, and assay background is significantly reduced. By comparing the his-tagged protein to a highly purified recombinant commercial protein, we were able to demonstrate that presence of a histidine tag does not stimulate a significant anti-his response in patients or normal controls. Furthermore, comparison to the purified protein indicates no significant interference from anti-nickel elements within samples, a possibility due to the presence of nickel sensitivity in approximately 13% of adults [14]. Methods for creating histadine tagged proteins and cell transfection are easy, fast and commercially available at modest cost. The method may potentially be used with any human tumor-associated antigen gene.

With the advent of high throughput technologies for antigen discovery we need high throughput methods for antigen evaluation. Success of the technique described here indicates that a similar system may be used to obtain clinical quality antigen specific for potentially any protein within a cDNA library, and thus development of a high-throughput, clinical grade assay is limited only by availability of the gene encoding the target protein.

## Abbreviations

SEREX: serological analysis of recombinant cDNA expression libraries; ELISA: enzyme-linked immunosorbant assay; CV: coefficient of variation; CHO: Chinese hamster ovary; OD: optical density; PBS: phosphate-buffered saline; BSA: bovine serum albumin; FCS: fetal calf serum; NGS: normal goat serum; SDS-PAGE: sodium dodecyl sulfate polyacrylamide gel electrophoresis; HRP: horseradish peroxidase.

## Authors' contributions

DMcN participated in study design and coordination, and created the transformed cell lines. VG validated and carried out the immunoassays and performed the statistical analysis. MLD initiated the project and participated in the design of the study and coordination, and helped to draft the manuscript. All authors read and approved the final manuscript.
